# Patient Preferences Regarding Surgical Treatment Methods for Symptomatic Uterine Fibroids

**DOI:** 10.1007/s43441-023-00525-1

**Published:** 2023-05-20

**Authors:** Olufemi Babalola, David Gebben, Michelle E. Tarver, Roopina Sangha, Jason Roberts, Veronica Price

**Affiliations:** 1 Patient Science & Engagement, Office of Strategic Partnerships and Technology Innovation, Center for Devices and Radiological Health, U.S. Food and Drug Administration, White Oak, Bldg. 66, Room 5574, Silver Spring, MD 20993 USA; 2 Center for Devices and Radiological Health, U.S. Food and Drug Administration, Silver Spring, MD USA; 3 Department of Obstetrics and Gynecology, JPS Health Network, Fort Worth, TX USA

**Keywords:** Patient preferences, Patient priorities, Benefit-risk assessment, Product development, Uterine fibroids, Best Worst Scaling

## Abstract

**Study Objective:**

The purpose of this study is to rank the factors that are most and least important to patients with symptomatic uterine fibroids when considering surgical treatment options.

**Materials and Methods:**

Using a best worst scaling (BWS) preference elicitation approach, participants completed an online survey to rank factors associated with fibroid surgical treatments. Survey content was based on a literature review and included the following factors: symptom relief; surgical complications; repeat treatment; recovery time; cosmetic effects; risk of spreading undiagnosed cancer; sexual outcomes; maintenance of child-bearing; continuation of menses; unpredictable menses; and location of procedure. Participants completed 11 BWS tasks. For each task, we presented participants with a subset of 5 factors from the possible 11, and participants chose the most important and least important factor. Participants’ responses were analyzed using conditional logistic regression to determine the relative importance of factors. Patient priorities were further explored via age and race.

**Results:**

285 respondents with symptomatic uterine fibroids (69 physician-confirmed and 216 self-reported) who had not undergone prior surgical treatment completed the survey. Respondents were enrolled from two clinical sites (clinical site cohort) and an online consumer panel (panel cohort). Both cohorts identified symptom relief, cancer risk, repeat treatment and complications as the most important factors in selecting surgical treatment options and location of procedure, return to normal activities after surgery, and cosmetic effects like presence of a scar after the surgical treatment as the least important factors. Of note, younger women (≤ 40) placed greater importance on the ability to have children after the procedure.

**Conclusion:**

Information regarding the factors most and least important to patients with symptomatic uterine fibroids might inform development and regulatory evaluation of new technologies and procedures. Study results may be useful in efforts to develop a set of outcomes to include in future fibroids clinical studies.

**Supplementary Information:**

The online version contains supplementary material available at 10.1007/s43441-023-00525-1.

## Introduction

Uterine fibroids (fibroids) are the most common type of benign abnormal growth in the uterus [1]. Although fibroids are non-cancerous, they may be associated with symptoms including heavy bleeding, pelvic pain, and infertility [2]. Fibroids most often occur in individuals of reproductive age. Common risk factors for fibroids include black race, premenopausal state, advanced reproductive age, increasing interval since last birth, and a positive family history [3–9].

Although hysterectomy is a definitive solution to symptomatic fibroids, there are minimally invasive surgical (incisional and non-incisional) alternatives that preserve the uterus and may offer comparable outcomes [10–12]. Examples include minimally invasive myomectomy, uterine artery embolization (UAE), magnetic resonance guided focused ultrasound (MRgFUS), and radiofrequency volumetric thermal ablation (RFVTA) for fibroids, and endometrial ablation (EA) for bleeding. Although we acknowledge the role that medical management can play in the treatment of benign fibroids, including hormonal intrauterine devices, our study focuses specifically on surgical options. There continues to be interest in new devices for minimally invasive treatment of uterine fibroids.

The evolving landscape of new technologies offering minimally invasive options for the surgical treatment of symptomatic fibroids present with distinct features including varying effectiveness and safety profiles. Individuals with symptomatic uterine fibroids live with their condition and have individualized perspectives on the relative value of benefits, acceptability of risks, and other attributes associated with various treatment options. Patients may perceive risks or benefits of a treatment differently than physicians or the regulators responsible for determining whether a new device can be marketed. Information on how patients prioritize the benefit-risk factors associated with procedures for symptomatic fibroids could be used to help inform regulatory and clinical decision-making [13–16].

To the best of our knowledge, there is currently no study that quantifies individuals’ preferences for surgical treatment options for their symptomatic fibroids by examination of the factors, including benefit-risk outcomes, that are most important to them. Best Worst Scaling (BWS) is a quantitative preference elicitation method for assessing individuals’ priorities by assessing the relative importance among a set of items [17]. The United States Food and Drug Administration (FDA) Center for Devices and Radiological Health (CDRH) issued guidance on patient preference information (PPI) that may be used in regulatory decision-making. The guidance includes recommendations for collecting PPI information including quantitative preference elicitation studies to understand patients’ benefit-risk preferences for medical devices [18].

The objective of this study is to use a BWS approach to quantify patient preferences by ranking the benefit-risk factors that are most and least important to patients with symptomatic fibroids as they consider surgical options. Additional analyses were performed to explore how preferences (factor rankings) differ by race and age.

## Materials and Methods

Our BWS study was designed and conducted according to research practices outlined by the International Society for Pharmacoeconomics and Outcomes Research (ISPOR) and FDA’s guidance on patient preference information [18, 19]. We first performed a literature search in PubMed to identify factors which impact preferences for surgical treatment of symptomatic fibroids. A detailed description of the literature review is provided elsewhere [20]. Eleven factors were identified from the review that were relevant to the regulatory context for surgical treatments of fibroids. Identified factors were as follows: symptom relief, surgical complications, repeat treatment, recovery time, cosmetic effects, risk of spreading undiagnosed cancer (cancer risk), sexual outcomes, maintenance of child-bearing capacity, continuation of menses, unpredictable menses, and location of procedure (hospital vs. office based). Table 1 provides a description of each factor used in the BWS.

**Table 1:** Factors in BWS Survey

Factor	Presentation of Factor in BWS Survey
Symptom relief	Relief from your symptoms of uterine fibroids after surgical treatment
Surgical complications	Concern about complications from the surgical treatment
Repeat treatment	Concern about the need for a repeat surgical treatment in future due to return of fibroid symptoms
Recovery time	The time it takes to return to work and normal activities after surgical treatment
Cosmetic effects	Avoiding a visible scar after surgical treatment
Cancer risk	Concern that there is existing cancer in the uterus that was not detected before surgery and might be spread by the surgical treatment
Sexual outcomes	Concern about decreased sexual desire and/or sexual satisfaction after surgical treatment
Maintenance of child-bearing capacity	Maintaining the ability to become pregnant and have children after surgical treatment
Continuation of menses	Retaining the ability to have menstrual periods after surgical treatment
Unpredictable menses	Having unpredictable menstrual periods after surgical treatment
Location of procedure	Having the surgical treatment at your doctor’s office (not the hospital)

### Survey Development

We designed a survey instrument using the BWS approach to identify which benefit-risk factors mattered most to individuals as they consider surgical treatment for their fibroids condition [21]. A BWS approach is grounded in random utility theory and has an advantage over traditional rating or other ranking scales of quantifying prioritization among relevant items, thereby increasing discriminative ability [22–24]. BWS presumes that respondents can choose between extreme items (best and worst, most and least, smallest and largest) from a set of three or more items presented in a choice task.

**Figure 1:**
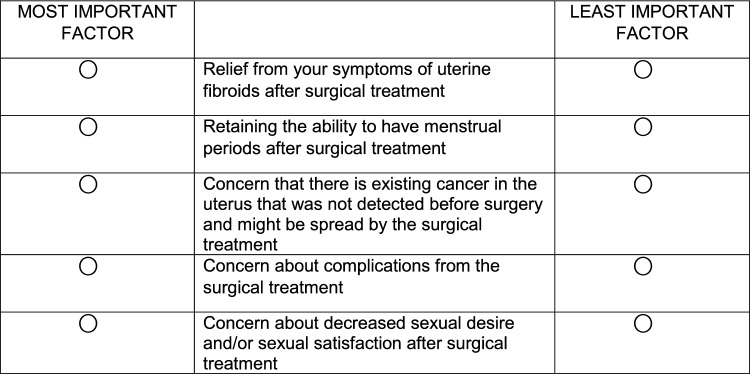
A Sample Best–Worst Choice Task. Suppose you have been asked by your doctor to consider some surgical treatment options for your uterine fibroids. From the choices below, please select the factor that is *most important* and select the *least important* factor when deciding about possible surgery. Please choose only one factor for each column.

For our study, we presented participants with 11 different BWS choice tasks. For each task, we presented participants with a subset of 5 factors from the possible 11, and participants chose the most and least important factors. The balanced incomplete block design (see Online Appendix) was used to develop the choice tasks to ensure desirable statistical properties like a fixed choice task size, and equal occurrence and co-occurrence of items across all choice tasks. Specifically, with this design each choice task contained 5 factors, each factor appeared five times and twice with another factor in the survey. Order of presentation of 11 choice tasks was randomized for each respondent to avoid ordering effects. Figure 1 is an example of a choice task.

In order to provide common understanding for all respondents, the survey provided a tutorial on the following: instructions on how to complete the survey, surgical treatment options for fibroids, and descriptions of the 11 factors identified in the literature review. In addition, the survey included questions on demographic characteristics and fibroid-related health experiences. Forced choice responses were enabled for all survey questions. Prior to the deployment of the online survey, the instrument was pretested in 5 face-to-face interviews with fibroid patients that met the study inclusion criteria from the University of Maryland obstetrics and gynecology clinic. During pre-testing we engaged patients to gain insight into their understanding of the survey content, identify unfamiliar terms, determine the relevance of the factors used in the BWS, and assess appropriateness of the survey length. The pretest interviews confirmed the relevance of the BWS factors and that the overall survey length was appropriate. Pretest participants provided feedback that was used to further refine the factor descriptions to ensure they were clear and easily understood.

### Data Collection

Participants eligible for inclusion were individuals with symptomatic fibroids who had not yet undergone a surgical treatment for fibroids and were not scheduled for a future surgery. Participants were required to be at least 22 years of age, living in the U.S., and able to read and answer the online survey in English.

Participants were initially enrolled from two clinical sites (“clinical site cohort”) which were Henry Ford Health System, Detroit, Michigan and JPS Health Network, Dallas, Texas. The clinical site cohort included individuals with a physician-confirmed diagnosis of symptomatic fibroids. Clinical staff at the sites identified eligible patients in clinic and discussed the study with them. Interested participants provided permission to the clinical sites for their contact details including name, phone number, and email address, which were shared with the FDA for survey administration and compensation purposes only. Interested participants that met the eligibility criteria at the clinical sites were invited by email to complete the survey. The online survey instrument for the clinical site cohort was programmed using Qualtrics software (Qualtrics, Provo, UT). Due to difficulty recruiting an adequate number of participants with a physician-confirmed diagnosis in a timely manner, we also engaged participants with self-reported diagnosis of symptomatic fibroids via a consumer panel (“panel cohort”). The panel cohort was recruited via a national healthcare online panel of U.S adults managed by Dynata, a survey research company (Dynata, Shelton, CT). The online panel constitutes of members who have volunteered to participate in health care research. For this respondent group, the online survey instrument was programmed by Dynata using the software Decipher (Forsta, New York, NY). The survey instruments were identical. However, additional screening questions for the panel cohort were designed to effectively screen out ineligible participants. (Sample screeners are included in an appendix).

The study was determined to be Institutional Review Board (IRB) exempt by the FDA and the local IRBs at the clinical sites. Informed consent documents were administered as part of the online survey to all respondents after passing the initial screener questions. Upon completion of the survey, all respondents were provided with a $25 gift card.

### Analysis

Data from the clinical site and panel cohorts were analyzed separately. Internet Protocol (IP) addresses were used to identify potential duplicate responses. Patients’ characteristics were described using descriptive statistics. Respondents who did not provide answers to the BWS choice questions or who completed the survey more than once were excluded from the final analysis.

In analyzing the BWS questions, we assumed sequential best–worst responses. This approach assumes that respondents first choose the factor that they think fits the criteria the best (i.e., most important) and then choose the worst (i.e., least important) from the remaining factors. This approach assumes that the choice of best is independent of the choice of worst, and that the choice of worst was conditional upon the choice of best. Each task was treated as two separate sub-tasks, where the most important factor was selected from 5 factors in each choice task and then the worst from the remaining 4 factors in the task. For data setup purposes, two separate datasets were created and later appended. In the “best” dataset, all 5 factors from each choice task were included; the independent choice variable was coded as one for the factor selected as most important and coded as zero for the 4 factors that were not selected as most important. In the worst dataset, the 4 factors for each choice task that were not selected as most important were included. The independent choice variable was coded as one for the factor selected as least important and as zero for the 3 factors not selected as least important. The variable for each factor was coded with a negative sign to reflect the reciprocal relationship between most and least important probabilities.

Effects coding was used to analyze the effect of each factor [25]. The choice of best and worst was described via a single dichotomous dependent variable. This approach was repeated for each respondent for each task. Using McFadden’s conditional logit, we modeled the binary choice variable on all effects-coded attributes (factors), where data were grouped by respondent, task, and type (i.e., best or worst) [26]. The regression results provide coefficients (relative importance/preference weights) for 10 factors. The coefficient for the 11th factor was estimated as the negative of the sum of the other 10 coefficients [27]. Furthermore, its standard error was estimated as the square root of the sum of the variance–covariance matrix from the initial regression [27]. The estimated coefficients represent a comparison of preferences for each factor relative to the reference (i.e., the grand mean). Unadjusted, the preference coefficients or weights take on both positive and negative values, with larger coefficients representing greater factor importance.

To further understand the impact of the various factors on fibroid surgical treatment decision-making, we analyzed the rankings stratified by respondent’s reported age and race. We categorized the age variable into two groups (≤ 40 and > 40 years of age; 40 years of age was selected as the demarcation because more than 90% of individuals give birth before the age of 40) [28]. The two groups (≤ 40 and > 40 years of age) are described as younger and old women. To check for differences in preferences via race, we stratified our sample by respondents that identified as Black or White and analyzed the data sets separately. All analyses were performed using Microsoft Excel and Stata 15 (StataCorp LP, College Station, TX, USA).

## Results

The online survey was administered to the clinical site cohort between November 2019 and January 2021. Email invitations were sent to 599 individuals identified from clinical sites to complete the study. Of the 161 individuals who clicked on the survey link, 83 (51.6%) were eligible and consented to participate with 69 respondents completing the survey. The online survey was administered to the Dynata panel cohort in May 2020. Dynata invited 13,754 individuals to participate in the study. Of the 939 panel participants who clicked on the survey link, 392 (41.8%) were eligible and consented to participate. The final sample included 216 respondents that completed the survey. There were no duplicate participant responses in the clinical and panel cohorts.

Table 2 presents the demographic and health characteristics for individuals from both the panel and clinical site cohorts who completed the surveys.

**Table 2:** Respondent Characteristics

Question	Clinical Site Cohort (*N* = 69)	Panel Cohort
		(*N* = 216)
How long ago were you diagnosed with fibroids? *n* (%)		
0–1 year	4 (5.8)	21 (9.7)
1–2 years	5 (7.3)	44 (20.4)
2–4	6 (8.7)	52 (24.1)
4 or more years	54 (78.3)	99 (45.83)
My fibroid symptoms have affected my quality of life? *n* (%)		
Strongly agree	21 (30.4)	62 (28.7)
Agree	17 (24.6)	104 (48.2)
Neutral	17 (24.6)	34 (15.7)
Disagree	13 (18.8)	13 (6.0)
Strongly disagree	1 (1.5)	3 (1.4)
Do you have a history of cancer? *n* (%)		
Yes	6 (8.7)	25 (11.6)
No	63 (91.3)	191 (88.4)
When was your last menstrual period? *n* (%)		
Less than 1 year ago	43 (62.3)	148 (68.5)
More than 1 year ago	26 (37.7)	68 (31.5)
What is your age?		
Mean	48.5	45
Median	49	44
Min, max	31, 65	22, 76
Age categories. *n* (%)		
≤ 40	14 (20.29)	87 (40.28)
> 40	55 (79.71)	129 (59.73)
Have children? *n* (%)		
Yes	47 (68.12)	120 (55.6)
No	22 (31.88)	96 (44.5)
Which of the following racial designations best describes you? *n* (%)		
American Indian or Alaska Native	0	1 (0.5)
Asian	2 (2.9)	12 (5.6)
Black or African American	47 (68.1)	49 (22.7)
Native Hawaiian	0	1 (0.5)
White	11 (15.9)	150 (69.4)
I would rather not answer	6 (8.7)	0
Other	3 (4.4)	3 (1.4)
What is the highest level of education you have completed? *n* (%)		
Less than high school	1 (1.5)	3 (1.4)
High school graduate or equivalent (such as GED)	8 (11.6)	20 (9.3)
Some college but no degree	11 (15.9)	31 (14.4)
Technical school	1 (1.5)	8 (3.7)
2-year college degree (Associate’s degree)	15 (21.7)	25 (11.6)
4-year college degree (such as a BA or BS)	11 (15.9)	75 (34.7)
Some graduate school but no degree	4 (5.8)	12 (5.6)
Graduate or professional degree (such as MBA, MS, MD, PhD)	15 (21.7)	42 (19.4)
I would rather not answer	3 (4.4)	0

*N* analytic sample, *n* number of respondents who answered specific item.

The clinical site and panel cohort reported a mean age of 48.5 and 45 years, respectively, and the majority of individuals in both samples (56% and 68%, respectively) had children. The clinical site cohort sample was largely comprised of Black individuals (68%) while most of the panel respondents were White (70%). The majority of respondents (87% and 89% of clinical site and panel respondents respectively) had more than a high school education.

A majority of respondents from the clinical site cohort (55%) and panel cohort (87%) noted that their fibroid symptoms affected their quality of life. In addition, 87% and 70% of individuals in the clinical site and panel cohorts, respectively, reported receiving their initial fibroids diagnosis more than 2 years ago. Most respondents reported no personal history of cancer (91.3%, 88.4% in clinical site and panel cohorts). Most of the respondents (62% and 69% in clinical site and panel cohorts) were pre-menopausal (last menstrual period < 1 year ago), whereas 38% and 32% in the same groups identified as post-menopausal (last menstrual period > 1 year ago).

**Figure 2:**
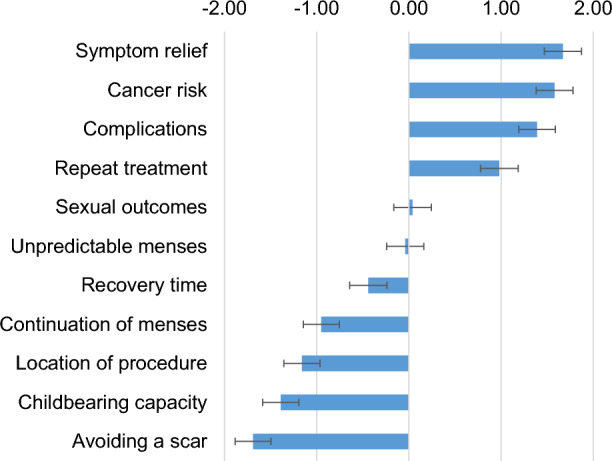
The Factor Rankings Among the Clinical Site Cohort. Bars denote the relative importance estimate for each factor. Error bars represent the 95% confidence intervals for the estimates. From the figure, on average, respondents noted symptom relief was the most important factor and avoiding a scar the least important.

**Figure 3:**
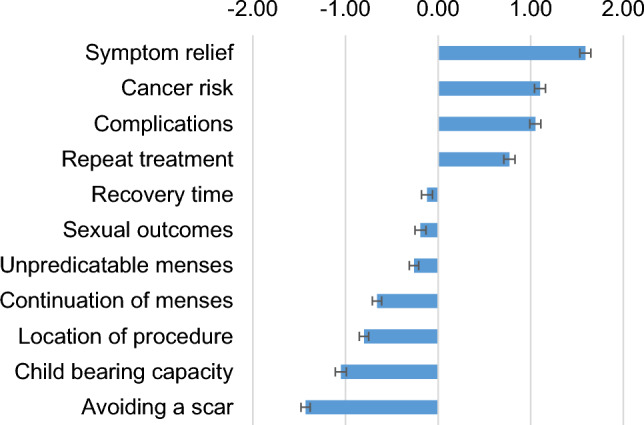
The Factor Rankings Among the Panel Cohort. Bars denote the relative importance estimate for each factor. Error bars represent the 95% confidence intervals for the estimates.

Figures 2 and 3 present the factor rankings based on the estimation of the regression model following the BWS exercise. Higher values (estimated preference coefficients) indicate a greater frequency of being selected in the choice experiment as the most important factor to fibroid surgical treatment choice. We observed similar preferences (factor rankings) between clinically validated and online panel respondents. Symptom relief was reported as the most important factor in surgical treatment decisions for symptomatic fibroids. Risk of cancer, surgical complications, and the need for repeat treatments were also factors noted to be very important when making fibroid surgical treatment decisions. Other factors like sexual outcomes, unpredictability of menses, and recovery time following a procedure were seen to be modestly/moderately important factors in the rankings. Maintenance of child-bearing capacity, continuation of menses, location of procedure, and cosmetic effects like the presence of an abdominal scar after surgical treatment were reported as less important.

To further understand the impact of the various factors on fibroid surgical treatment decision-making, we analyzed the rankings stratified by respondent’s reported age and race (Tables 3 and 4).

**Table 3:** Best–Worst Scaling Estimates and Rankings of Relative Importance (Preference) Weights of Attributes for the Overall Clinical Sample, and by Race and Age

Attributes (Factors)	Overall (*n* = 69)		Race				Age			
			White (*n* = 11)		Black (*n* = 47)		≤ 40 (*n* = 14)		> 40 (*n* = 55)	
	Estimate (95% CI)	Rank	Estimate (95% CI)	Rank	Estimate (95% CI)	Rank	Estimate (95% CI)	Rank	Estimate (95% CI)	Rank
Symptom relief	1.67 (1.47, 1.87)	1st	2.26 (1.72, 2.80)	1st	1.55 (1.31, 1.80)	2nd	0.97 (0.51, 1.43)	4th	1.95 (1.72, 2.19)	1st
Repeat treatment	0.98 (0.77, 1.18)	4th	0.71 (0.17, 1.25)	4th	1.13 (0.88, 1.37)	4th	0.42 (− 0.09, 0.92)	5th	1.20 (0.97, 1.44)	4th
Maintenance of childbearing capacity	− 1.39 (− 1.59, − 1.18)	10th	− 1.92 (− 2.45, − 1.40)	11th	− 1.26 (− 1.51, − 1.01)	10th	1.65 (1.19, 2.1)	2nd	− 2.02 (− 2.25, − 1.78)	11th
Continuation of menses	− 0.95 (− 1.15, − 0.75)	8th	− 1.32 (− 1.83, − 0.81)	9th	− 0.79 (− 1.03, − 0.55)	8th	− 0.56 (− 1.02, − 0.09)	8th	− 1.17 (− 1.40, − 0.94)	9th
Unpredictable menses	− 0.04 (− 0.24, 0.17)	6th	− 0.17 (− 0.69, 0.36)	6th	0.01 (− 0.24, 0.25)	5th	− 0.27 (− 0.75, 0.22)	7th	0.01 (− 0.22, 0.25)	6th
Risk of cancer	1.58 (1.38, 1.78)	2nd	1.77 (1.25, 2.29)	2nd	1.62 (1.38, 1.86)	1st	2.01 (1.55, 2.46)	1st	1.60 (1.37, 1.84)	2nd
Recovery time	− 0.44 (− 0.64, − 0.24)	7th	0.17 (− 0.36, 0.71)	5th	− 0.64 (− 0.89, − 0.40)	7th	− 1.15 (− 1.61, − 0.69)	9th	− 0.31 (− 0.54, − 0.07)	7th
Surgical complications	1.39 (1.19, 1.59)	3rd	1.49 (0.97, 2.01)	3rd	1.40 (1.16, 1.64)	3rd	1.33 (0.88, 1.79)	3rd	1.52 (1.29, 1.75)	3rd
Absence of abdominal scar	− 1.69 (− 1.89, − 1.50)	11th	− 1.65 (− 2.16, − 1.15)	10th	− 1.78 (− 2.02, − 1.54)	11th	− 2.15 (− 2.63, − 1.68)	11th	− 1.78 (− 2.01, − 1.55)	10th
Sexual outcomes	0.04 (− 0.16, 0.25)	5th	− 0.19 (− 0.72, 0.35)	7th	− 0.08 (− 0.33, 0.17)	6th	− 0.17 (− 0.66, 0.32)	6th	0.09 (− 0.15, 0.33)	5th
Location of procedure	− 1.16 (− 1.35, − 0.96)	9th	− 1.15 (− 1.67, − 0.62)	8th	− 1.16 (− 1.40, − 0.92)	9th	− 2.08 (− 2.72, − 1.44)	10th	− 1.12 (− 1.35, − 0.88)	8th

Estimate represents respondents’ average relative importance (preference) weights for a given factor.

*CI* confidence interval.

**Table 4:** Best–Worst Scaling Estimates and Rankings of Relative Importance (Preference) Weights of Attributes for the Overall Panel Sample, and by Race and Age

Attributes (Factors)	Overall (*n* = 216)		Race				Age			
			White (*n* = 150)		Black (*n* = 49)		≤ 40 (*n* = 87)		> 40 (*n* = 129)	
	Estimate (95% CI)	Rank	Estimate (95% CI)	Rank	Estimate (95% CI)	Rank	Estimate (95% CI)	Rank	Estimate (95% CI)	Rank
Symptom relief	1.59 (1.49, 1.70)	1st	1.74 (1.61, 1.87)	1st	1.25 (1.03, 1.47)	1st	1.28 (1.11, 1.44)	1st	1.97 (1.82, 2.12)	1st
Repeat treatment	0.77 (0.66, 0.88)	4th	0.74 (0.60, 0.87)	4th	0.80 (0.57, 1.02)	4th	0.47 (0.30, 0.64)	5th	1.11 (0.96, 1.26)	4th
Maintenance of childbearing capacity	− 1.05 (− 1.16, − 0.94)	10th	− 1.42 (− 1.56, − 1.29)	10th	0.07 (− 0.16, 0.31)	5th	0.48 (0.30, 0.65)	4th	− 2.10 (− 2.26, − 1.95)	11th
Continuation of menses	− 0.66 (− 0.77, − 0.56)	8th	− 0.79 (− 0.92, − 0.66)	9th	− 0.38 (− 0.60, − 0.16)	8th	− 0.07 (− 0.24, 0.10)	6th	− 1.19 (− 1.34, − 1.04)	9th
Unpredictable menses	− 0.26 (− 0.37, − 0.15)	7th	− 0.26 (− 0.39, − 0.13)	7th	− 0.29 (− 0.51, − 0.07)	7th	− 0.23 (− 0.40, − 0.07)	7th	− 0.32 (− 0.47, − 0.17)	7th
Risk of cancer	1.10 (0.99, 1.20)	2nd	1.18 (1.05, 1.31)	2nd	0.87 (0.65, 1.09)	3rd	0.82 (0.65, 0.99)	2nd	1.42 (1.27, 1.57)	3rd
Recovery time	− 0.12 (− 0.22, − 0.01)	5th	0.01 (− 0.12, 0.14)	5th	− 0.40 (− 0.62, − 0.18)	9th	− 0.47 (− 0.63, − 0.30)	9th	0.15 (0.00, 0.30)	5th
Surgical complications	1.05 (0.94, 1.15)	3rd	1.10 (0.97, 1.23)	3rd	0.88 (0.66, 1.09)	2nd	0.68 (0.51, 0.84)	3rd	1.43 (1.28, 1.57)	2nd
Absence of abdominal scar	− 1.43 (− 1.53, − 1.32)	11th	− 1.41 (− 1.54, − 1.28)	11th	− 1.54 (− 1.76, − 1.32)	11th	− 1.57 (− 1.74, − 1.40)	11th	− 1.51 (− 1.66, − 1.37)	10th
Sexual outcomes	− 0.19 (− 0.30, − 0.08)	6th	− 0.17 (− 0.31, 0.04)	6th	− 0.26 (− 0.48, − 0.04)	6th	− 0.35 (− 0.51, − 0.18)	8th	− 0.12 (− 0.27, 0.03)	6th
Location of procedure	− 0.80 (− 0.91, − 0.69)	9th	− 0.71 (− 0.84, − 0.58)	8th	− 1.01 (− 1.23, − 0.79)	10th	− 1.02 (− 1.19, − 0.86)	10th	− 0.83 (− 0.98, − 0.67)	8th

Estimate represents respondents’ average relative importance (preference) weights for a given factor.

*CI* confidence interval.

Younger individuals in both the panel and clinical site cohorts identified symptom relief, cancer risk, complications, and maintenance of child-bearing capacity as the most important factors in selecting surgical treatment options. Repeat treatment, sexual outcomes, and unpredictable menses were also seen as important. Least important factors were location of procedure, recovery time, and cosmetic effects. Older individuals in both samples also identified symptom relief, cancer risk, complications, and repeat treatment as the most important factors. Sexual outcomes, and unpredictable menses were also deemed important. Older individuals in both samples deemed maintenance of child-bearing capacity and cosmetic effects as the least important factors in selecting surgical treatment options.

Overall, no differences in the rankings were observed by race. Symptom relief, cancer risk, complications, and repeat treatment were seen as the most important factors among White and Black respondents in both the panel and clinical site cohorts. In the panel sample, location of procedure and cosmetic effects were seen to be the least important factors to surgical treatment decision-making among Black respondents. Although White respondents also ranked cosmetic effects as least important, childbearing capacity was ranked low as well. In contrast, for the clinical site cohort, maintenance of childbearing capacity and cosmetic effects are the least important factors for both White and Black respondents.

## Discussion

Our study provides insights to women’s preferences regarding surgical treatment options for uterine fibroids, in general, and how these preferences may or may not be associated with race or age. Study participants in both samples (‘clinical site cohort’ and ‘panel cohort’) identified symptom relief and cancer risk as the two most important factors in choosing surgical treatments. The need for repeat treatment and treatment complications followed closely as very important factors in selecting surgical treatment options. These preferences were the same across race and age. Sexual dysfunction and unpredictability of periods were also identified by patients as important in their surgical treatment decision-making process, though less so. Cosmetic effects such as presence of a scar after the surgical treatment and location of procedure were identified as least important. After stratifying the clinical site and panel cohorts by age, younger individuals (≤ 40) placed greater importance on the ability to have children after the procedure.

Based on a national survey of women aged 29–59, uterine fibroids are estimated to affect 70% of white individuals and > 80% of black individuals by age 50 [3].There are numerous treatment options for symptomatic fibroids which range from medical therapies to less invasive surgical treatments, to invasive surgical treatments (e.g., hysterectomy). Studies have shown that black or African American (AA) individuals are more likely to undergo hysterectomy when compared to white individuals [4, 29]. In the publication by Stewart et al., the authors found that AA individuals were more concerned that their fibroids would affect their ability to have children when compared to white individuals, were more likely to value a treatment option that preserved their ability to get pregnant, and were more likely to view a uterine-preserving treatment option as very important when compared to white individuals [30]. While the reasons for an increased rate of hysterectomy in the AA population are likely multi-factorial, this does raise a question as to whether treatment decisions are aligned with patient preferences.

As noted by Marsh et. Al., there has been little research that has elucidated patient’s perspectives on fibroid treatment decisions, in spite of the fact that patient-centered care has been highlighted as a necessary component for improving the quality of health care [31]. There have been publications examining patient preference and treatment choice [32, 33]. However, no information was provided on how these preferences were derived. In addition, these studies did not examine whether age or race may have influenced preferences. While our study provided some insight into how patient preferences may differ by race and age, future research is needed to further build the understanding of preferences by race, age, and other demographic and health characteristics.

Understanding these preferences can aid in shared decision-making. However, our study also highlights the need for collection of meaningful clinical data on the various surgical treatment options for uterine fibroids, as well as identification of important focus areas in development of treatments. In particular, while clinical studies typically evaluate reduction in menstrual bleeding and need for retreatment which is consistent with outcomes that patients find important, the results of our study suggest that clinical trials should consider prioritizing other outcomes related to symptom relief (e.g., pelvic pressure, pain), cancer risk, sexual dysfunction, and unpredictability of periods. As noted in Tran et.al., the lack of standardization of outcomes and instruments in clinical studies as well as the inconsistent measurement of outcomes of importance to patients with uterine fibroids hampers the ability of patients and other stakeholders to make informed choices [34]. This has led to calls for research into identifying a core outcome set (COS) that measures outcomes of importance to patients. Identifying and including COS in studies of uterine fibroid surgical treatments will help assure that the data generated by research is relevant to patients, providers, regulators, and other stakeholders. Additionally, our study highlights that younger individuals have a stronger preference for fertility sparing surgical treatments. There are very few medical devices that are currently FDA-cleared or approved as fertility-sparing treatment of uterine fibroids [35]. Therefore, this may be an area where development of devices would be impactful. The benefit-risk assessment for new surgical fibroid treatments may be meaningfully informed by patient preferences [36, 37].

This study has several limitations to note. As with all stated preference research, this study elicits preferences in hypothetical scenarios that may not fully represent the clinical context. While patients underwent pretesting to confirm the relevance of the various factors, decisions made in the survey may not fully replicate decisions made in a clinical setting where other considerations may come into play. It is possible that excluding respondents who did not provide survey responses from analysis may result in bias on the population-level estimates if there was a systematic lack of representativeness. We also acknowledge that results from the panel cohort may be affected by volunteer bias due to the panel recruitment method. One cannot rule out the possibility that any differences in results between the clinical and panel cohorts may be due to different baseline characteristics (including race) of each data source. However, data from the clinical and panel cohorts were analyzed separately. The preference results from each cohort were similar. In addition, we further analyzed preferences for each data source stratified by age and race, and we also noticed that the preference results for both cohorts were largely similar. When exploring preference heterogeneity by race, our analysis was limited to black and white individuals, thus limiting inferences that can be made for other races. In addition, the clinical site cohort was of limited sample size and not geographically representative of people living with fibroids in the United States (US). However, the panel cohort was larger, recruited nationally and likely more geographically representative of US fibroid patients. More geographic data collection is needed to confirm generalizability of the results.

The strengths of this study include the examination of patient preference in a quantitative manner that allowed participants to convey those issues that are most and least important to them from a well-defined list of eleven factors. An additional strength is the size and diversity of the study population which allowed us to examine preferences by age (≤ 40 years and > 40 years) and by Black or White race. Lastly, by including clinically confirmed patients with self-reported patients, our study was able to show how different sources of participants may yield similar inferences.

## Conclusion

Individuals diagnosed with symptomatic uterine fibroids have several current and emerging surgical treatment options for their condition. The surgical treatment options include varying minimally invasive methods utilizing distinct device design features and principles of operation (e.g., UAE, MRgFUS, RFVTA) with varying benefit-risk profiles. The findings from this study offer insights into how individuals prioritize the benefits, risks, and other outcomes when considering surgical treatment options for their condition. The results of this study can provide insights to guide development and regulatory evaluation of new technologies and procedures.

## Supplementary Information

Below is the link to the electronic supplementary material.

Supplementary file1 (DOCX 110 kb).

## Data Availability

All relevant data are within the manuscript and its supporting Information files.
