# Supplementary material for: Patient Preferences Regarding Surgical Treatment Methods for Symptomatic Uterine Fibroids
Source: Ther Innov Regul Sci. 2023 May 20;57(5):976–86. doi: 10.1007/s43441-023-00525-1 (PMC10400705; doi:10.1007/s43441-023-00525-1)
Supplement: Supplementary file 1 — Supplementary file1 (DOCX 110 kb). [file 43441_2023_525_MOESM1_ESM.docx]

**APPENDIX**

**BIBD Design**


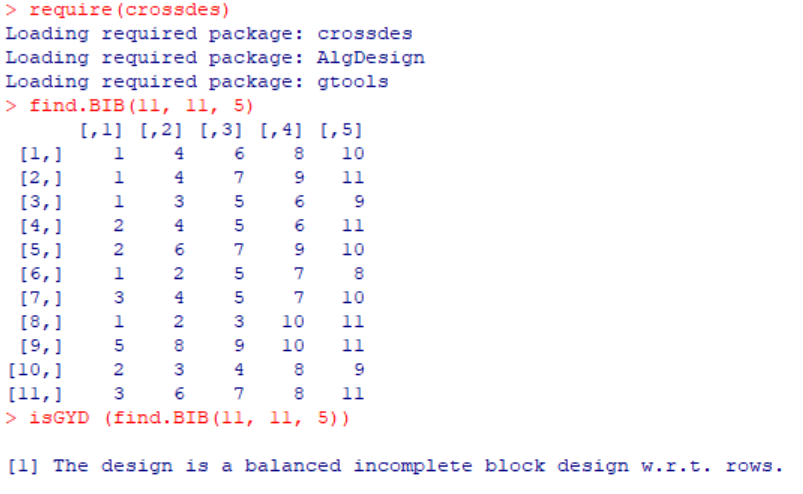


**Screener: Panel cohort**

S1. Are you 22 years of age or older? (please select one answer)

Yes [Continue]

No [Ineligible/Terminate]

S2. Please indicate all the conditions that you have been diagnosed with. [Multi- select]

- 1. High blood pressure
  2. Uterine Fibroids [Ineligible if not selected]
  3. Breast cancer
  4. Heart disease
  5. Ovarian / cervical cancer
  6. Endometriosis
  7. Lupus
  8. None of the above *[Anchor Exclusive]* Terminate

S3. Please select any of the symptoms you currently experience due to your Uterine Fibroid. (Please select all that apply)

1. Longer, more frequent, or heavy menstrual periods
2. Severe menstrual pain (cramps)
3. Vaginal bleeding at times other than menstruation
4. Anemia (from blood loss)
5. Pain in the abdomen or lower back
6. Pain during sex
7. Difficulty urinating or frequent urination
8. Constipation
9. Rectal pain
10. Difficult bowel movements
11. Infertility
12. None of the above *[Anchor Exclusive]*
13. Don’t know/ not sure *[Anchor Exclusive]*

Ineligible if l or M are selected.

S4. Which, if any, of the treatments have you received for your uterine fibroids?

(Please select all that apply)

Medications

Intrauterine Devices (IUD)

Contraceptive Pills and Progestational Agents

Surgery (Myomectomy, Hysterectomy, Magnetic Resonance Imaging - Guided Ultrasound surgery, Endometrial ablation, Uterine Artery Embolization.

1. None of the above

[Ineligible if D is selected]

S5. Are you scheduled for a surgical treatment for your uterine fibroids?

(Please select one answer)

Yes [Ineligible, end survey]

No [Continue]

Don’t know or not sure [Ineligible, end survey]

**Screener: Clinical site cohort**

**[Screening Questions]**

S1. Are you 22 years of age or older?

Yes [Continue]

No [Ineligible, end survey]

S2. Has a doctor ever told you that you have uterine fibroids?

Yes [Continue]

No [Ineligible, end survey]

Don’t know or not sure [Ineligible, end survey]

S3. Do you currently experience any of the symptoms below due to your uterine fibroid condition?

- Longer, more frequent, or heavy menstrual periods
- Severe menstrual pain (cramps)
- Vaginal bleeding at times other than menstruation
- Anemia (from blood loss)
- Pain in the abdomen or lower back
- Pain during sex
- Difficulty urinating or frequent urination
- Constipation
- Rectal pain
- Difficult bowel movements
- Infertility

Yes [Continue]

No [Ineligible, end survey]

Don’t know or not sure [Ineligible, end survey]

S4. Have you had a surgical treatment for your uterine fibroids condition?

Yes [Ineligible, end survey]

No [Continue]

Don’t know or not sure [Ineligible, end survey]

S5. Are you scheduled for a surgical treatment for your uterine fibroids?

Yes [Ineligible, end survey]

No [Continue]

Don’t know or not sure [Ineligible, end survey]
